# Greater Invasion and Persistence of *mcr-1-*Bearing Plasmids in Escherichia coli than in Klebsiella pneumoniae

**DOI:** 10.1128/spectrum.03223-22

**Published:** 2023-03-28

**Authors:** Yi-Yun Liu, Xiao-Qing Zhu, Sue C. Nang, Haoliang Xun, Luchao Lv, Jun Yang, Jian-Hua Liu

**Affiliations:** a National Risk Assessment Laboratory for Antimicrobial Resistance of Animal Original Bacteria, Guangdong Provincial Key Laboratory of Veterinary Pharmaceutics Development and Safety Evaluation, South China Agricultural University, Guangzhou, China; b Guangdong Laboratory for Lingnan Modern Agriculture, Guangzhou, China; c Biomedicine Discovery Institute, Monash University, Clayton, Victoria, Australia; d Department of Microbiology, School of Biomedical Sciences, Monash University, Clayton, Victoria, Australia; The Pirbright Institute

**Keywords:** colistin, resistance, *mcr-1*, plasmids, *Escherichia coli*, *Klebsiella pneumoniae*

## Abstract

The emergence of the plasmid-borne polymyxin resistance gene *mcr-1* threatens the clinical utility of last-line polymyxins. Although *mcr-1* has disseminated to various *Enterobacterales* species, the prevalence of *mcr-1* is the highest among Escherichia coli isolates while remaining low in Klebsiella pneumoniae. The reason for such a difference in prevalence has not been investigated. In this study, we examined and compared the biological characteristics of various *mcr-1* plasmids in these two bacterial species. Although *mcr-1*-bearing plasmids were stably maintained in both E. coli and K. pneumoniae, the former presented itself to be superior by demonstrating a fitness advantage while carrying the plasmid. The inter- and intraspecies transferability efficiencies were evaluated for common *mcr-1*-harboring plasmids (IncX4, IncI2, IncHI2, IncP, and IncF types) with native E. coli and K. pneumoniae strains as donors. Here, we found that the conjugation frequencies of *mcr-1* plasmids were significantly higher in E. coli than in K. pneumoniae, regardless of the donor species and Inc types of the *mcr-1* plasmids. Plasmid invasion experiments revealed that *mcr-1* plasmids displayed greater invasiveness and stability in E. coli than in K. pneumoniae. Moreover, K. pneumoniae carrying *mcr-1* plasmids showed a competitive disadvantage when cocultured with E. coli. These findings indicate that *mcr-1* plasmids could spread more easily among E. coli than among K. pneumoniae isolates and that *mcr-1* plasmid-carrying E. coli has a competitive advantage over K. pneumoniae, leading to E. coli being the main *mcr-1* reservoir.

**IMPORTANCE** As infections caused by multidrug-resistant “superbugs” are increasing globally, polymyxins are often the only viable therapeutic option. Alarmingly, the wide spread of the plasmid-mediated polymyxin resistance gene *mcr-1* is restricting the clinical utility of this last-line treatment option. With this, there is an urgent need to investigate the factors contributing to the spread and persistence of *mcr-1*-bearing plasmids in the bacterial community. Our research highlights that the higher prevalence of *mcr-1* in E. coli than in K. pneumoniae is attributed to the greater transferability and persistence of *mcr-1*-bearing plasmid in the former species. By gaining these important insights into the persistence of *mcr-1* in different bacterial species, we will be able to formulate effective strategies to curb the spread of *mcr-1* and prolong the clinical life span of polymyxins.

## INTRODUCTION

Multidrug-resistant (MDR) bacteria have become one of the largest global health threats, with a reduced number of effective antimicrobial agents against them (1). Plasmids play a key role in the evolution of MDR bacteria by acting as vehicles for transferring antimicrobial resistance genes. Of concern, *Enterobacterales* such as Klebsiella pneumoniae are considered crucial nosocomial pathogens associated with high rates of antibiotic resistance (2, 3), and carbapenem-resistant *Enterobacterales* (CRE) have been identified by the World Health Organization (WHO) as critical-priority pathogens that urgently need the development of new drugs (4). Due to limited treatment options, polymyxins (i.e., colistin and polymyxin B), belonging to an “old” class of antibiotics, were revived as last-resort agents against infections caused by CRE. Unfortunately, increasing frequencies of polymyxin-resistant bacteria are emerging (5); of note, the global dissemination of a plasmid-mediated colistin resistance gene (*mcr-1*) is threatening the role of polymyxins as a last-line treatment (6, 7).

The *mcr-1* gene is carried mainly by plasmids such as those of the IncX4, IncI2, IncHI2, IncP, IncFII, and IncFIB plasmid types, and the first three plasmid types represent the most prevalent epidemic vectors (8, 9). *mcr-1*-bearing plasmids have been spreading among various Gram-negative species (Escherichia coli, K. pneumoniae, Klebsiella oxytoca, Salmonella enterica, Cronobacter sakazakii, *Aeromonas* spp., *Moraxella* spp., and Enterobacter spp.) in diverse geographical locations (9, 10). Moreover, epidemiological data have shown that the prevalence of *mcr-1* is significantly higher in E. coli than in K. pneumoniae (11, 12); however, the reason for such a difference remains unclear.

The persistence of resistance plasmids in a bacterial population is usually considered to be associated with (i) plasmid maintenance during bacterial replication, (ii) fitness costs to the host, or (iii) plasmid transmission via conjugation (13, 14). The expression of *mcr-1* has been reported to produce a toxic effect on bacteria and impose fitness costs on both E. coli and K. pneumoniae (15–17). Notwithstanding, the persistence of *mcr-1*-bearing plasmids in bacterial populations is promoted by the strict control of the plasmid copy number and effective plasmid conjugation (18, 19). Besides, it has been suggested that the plasmid types in different bacterial species vary as a result of plasmid-host interactions (20, 21). Therefore, we speculate that the significant differences in the prevalences of *mcr-1* between E. coli and K. pneumoniae may be related to differences in the stabilities, conjugation efficiencies, and fitness costs of *mcr-1*-bearing plasmids in these two bacterial species. We thus compared the biological characteristics, including the fitness, transferability, and stability, of native *mcr-1* plasmids in E. coli and K. pneumoniae. Our results reveal that *mcr-1*-bearing plasmids transfer more readily and are more stably maintained in E. coli than in K. pneumoniae.

## RESULTS

### Comparison of the stabilities of *mcr-1*-carrying plasmids in E. coli and K. pneumoniae.

As *mcr-1* has been commonly identified in IncX4- and IncI2-type plasmids (9, 10), four native plasmids harboring *mcr-1*, isolated from E. coli (pHNGDE4P170 [IncX4] and pHNSHP45 [IncI2]) and K. pneumoniae (pHNAHM7C25I [IncX4] and pHNBJ7H48 [IncI2]), were chosen for the stability assay in this study. These plasmids were introduced into E. coli C600 and K. pneumoniae ATCC 13883 by electroporation, and the transformants were used for the stability assay. All native plasmids were found to be stably maintained in E. coli C600 and K. pneumoniae ATCC 13883 for 35 days of passage in the absence of selection pressure (see Table S1 in the supplemental material). These results suggest that there is no difference in the stabilities of these native *mcr-1*-carrying plasmids among the E. coli and K. pneumoniae strains used in this study.

### Comparison of the fitness costs of *mcr-1*-bearing plasmids in E. coli and K. pneumoniae.

**FIG 1:**
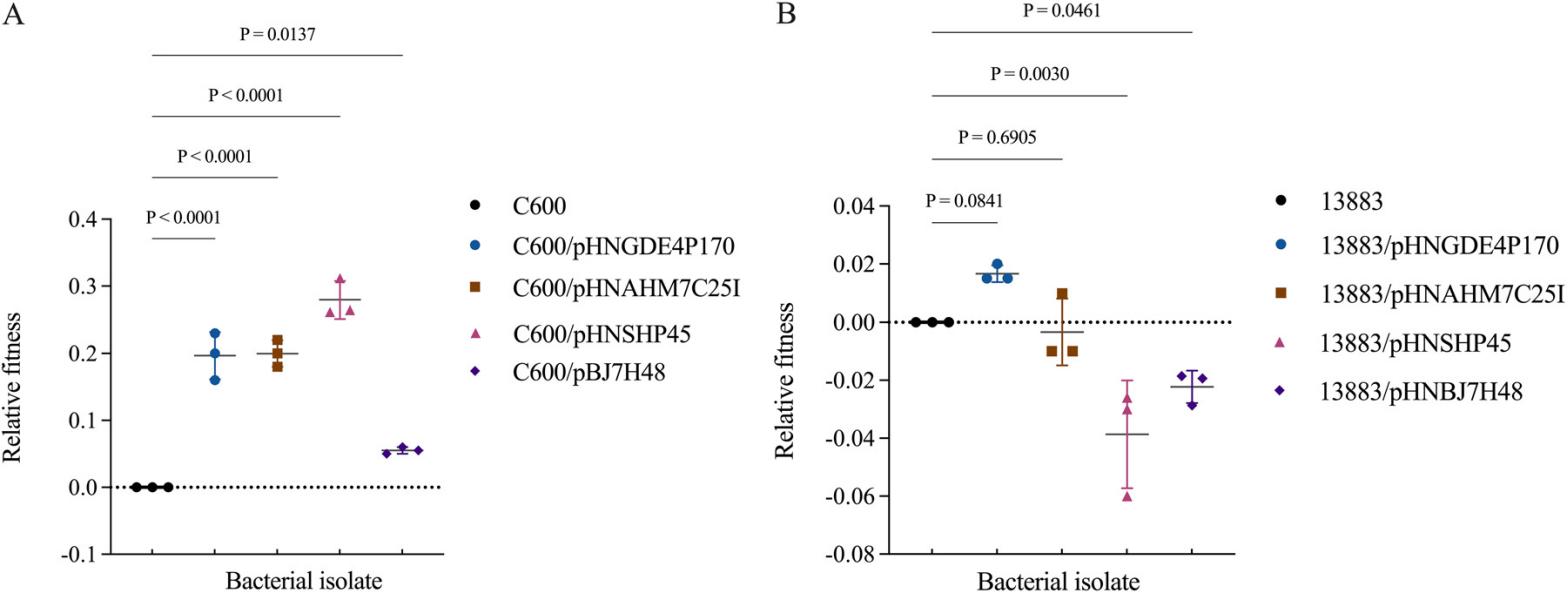
Effect of *mcr-1* plasmids on the fitness of E. coli C600 (A) and K. pneumoniae ATCC 13883 (B). E. coli C600, K. pneumoniae ATCC 13883, or their corresponding transconjugants carrying *mcr-1* plasmids (pHNGDE4P170 and pHNAHM7C25I [IncX4]; pHNSHP45 and pHNBJ7H48 [IncI2]) were competed against the reference strain E. coli DH5α-GFP *in vitro*, separately (*n *= 3; means ± standard deviations [SD]). The differences in relative fitness were determined by one-way analysis of variance with Benjamini-Hochberg correction.

The biological costs of *mcr-1*-bearing IncX4-type plasmids (pHNGDE4P170 or pHNAHM7C25I) or IncI2-type plasmids (pHNSHP45 or pHNBJ7H48) in E. coli and K. pneumoniae strains were investigated in a competition assay with E. coli DH5α-GFP as the reference strain. All four E. coli strains carrying different *mcr-1* plasmids demonstrated higher selection coefficients than the reference strain, suggesting that *mcr-1*-positive E. coli showed an increased fitness in comparison to the control strain (C600/pHNGDE4P170, *P < *0.0001; C600/pHNAHM7C25I, *P < *0.0001; C600/pHNSHP45, *P < *0.0001; C600/pBJ7H48, *P *= 0.0137 [by one-way analysis of variance {ANOVA} with Benjamini-Hochberg correction]) (Fig. 1A). For K. pneumoniae ATCC 13883, a higher selection coefficient was noted for the strain carrying pHNGDE4P170, an IncX4-type *mcr-1* plasmid (*P *= 0.0841 [by ANOVA with Benjamini-Hochberg correction]), while equivalent fitness was displayed by the strain with another IncX4-type *mcr-1* plasmid (pHNAHM7C25I) (*P *= 0.6905 [by ANOVA with Benjamini-Hochberg correction]) (Fig. 1B). Notably, lower fitness was illustrated by K. pneumoniae ATCC 13883 with IncI2-type *mcr-1* plasmids (ATCC 13883/pHNSHP45, *P *= 0.003; ATCC 13883/pHNBJ7H48, *P *= 0.0461 [by ANOVA with Benjamini-Hochberg correction]) (Fig. 1B). Overall, *mcr-1*-bearing plasmids conferred a fitness advantage on the E. coli host, whereas a slight fitness cost was imposed on K. pneumoniae.

### Comparison of the efficiencies of transfer of *mcr-1*-carrying plasmids into E. coli and K. pneumoniae.

**FIG 2:**
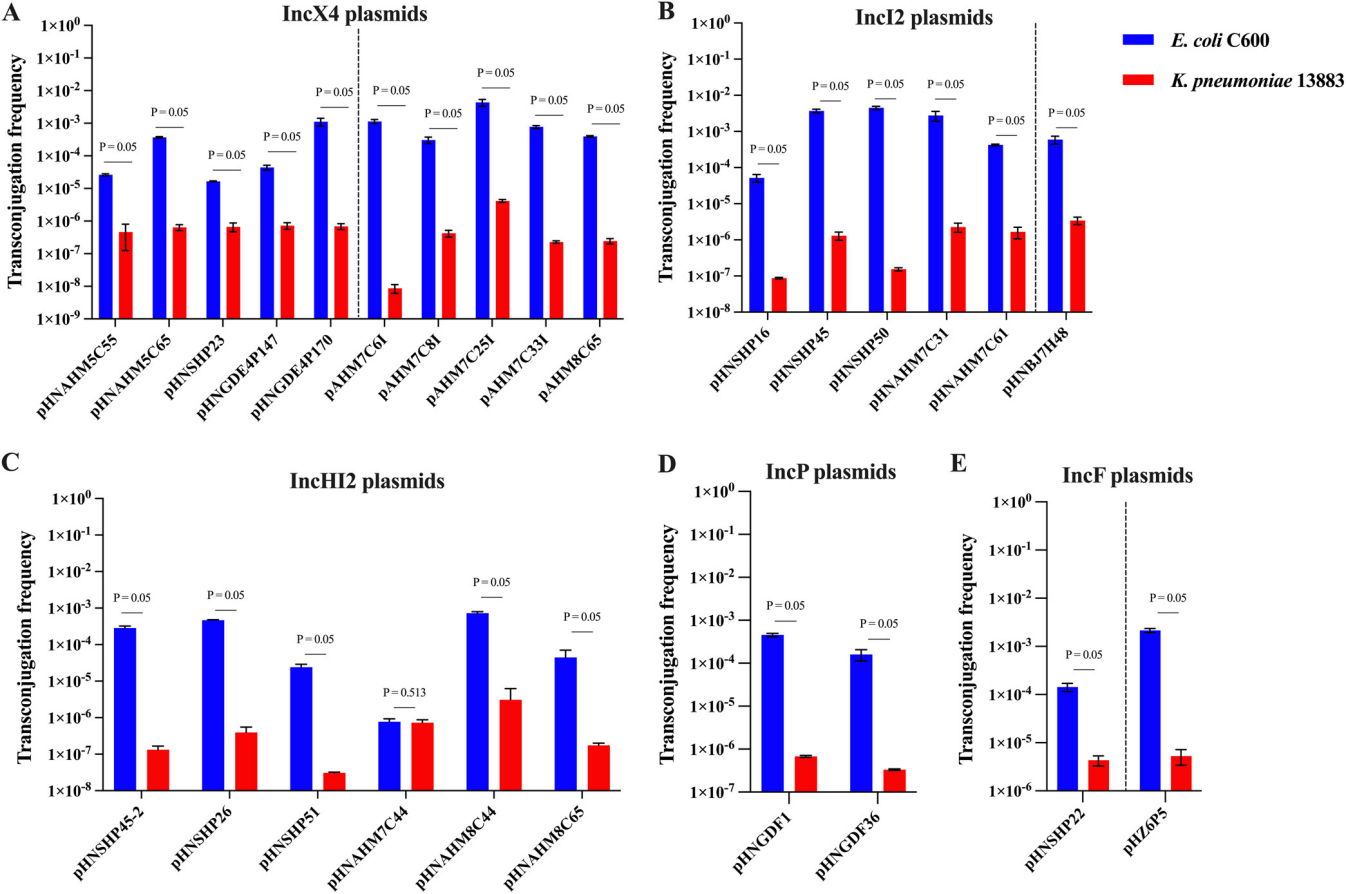
Frequencies of conjugation of *mcr-1* plasmids in E. coli and K. pneumoniae. (A, B, and E) *mcr-1* plasmids natively originating from E. coli and K. pneumoniae (left and right of the dotted lines, respectively) (*n *= 3; means ± SD). (C and D) IncHI2 and IncP plasmids natively originating from E. coli (*n *= 3; means ± SD). The difference in conjugation frequencies between two groups was determined by a nonparametric Mann-Whitney U test.

The inter- and intraspecies transferability efficiencies were evaluated for native *mcr-1* plasmids of the IncX4, IncI2, IncHI2, IncP, and IncF types in both E. coli and K. pneumoniae (Fig. 2A, B, and E). With E. coli C600 as the recipient strain, the *mcr-1* plasmids were transferred from native E. coli at frequencies of ~10^−5^ to 10^−3^ and from native K. pneumoniae at frequencies of ~10^−4^ to 10^−3^. With K. pneumoniae as the recipient strain, the *mcr-1* plasmids were transferred from native E. coli at frequencies of ~10^−7^ to 10^−5^ and from native K. pneumoniae at frequencies of ~10^−8^ to 10^−5^. The conjugation frequencies were ~10^−5^ to 10^−3^ for IncX4-type, ~10^−4^ to 10^−3^ for IncI2-type, ~10^−6^ to 10^−4^ for IncHI2-type, ~10^−4^ to 10^−3^ for IncP-type, and ~10^−4^ to 10^−3^ for IncF-type *mcr-1* plasmids in E. coli C600 (Fig. 2), while for the recipient K. pneumoniae ATCC 13883, the conjugation frequencies were ~10^−8^ to 10^−5^ for IncX4-type, ~10^−7^ to 10^−6^ for IncI2-type, ~10^−8^ to 10^−6^ for IncHI2-type, ~10^−7^ to 10^−6^ for IncP-type, and ~10^−6^ to 10^−5^ for IncF-type *mcr-1* plasmids (Fig. 2).

These results indicate that the frequencies of conjugation of individual *mcr-1* plasmids (IncX4, IncI2, IncHI2, IncP, and IncF types) were generally higher when E. coli C600 was employed as the recipient strain (~10^−5^ to 10^−3^) than when K. pneumoniae ATCC 13883 was used (~10^−8^ to 10^−6^) (Z = −1.964; *P *= 0.05 [by a Mann-Whitney U test]), with the exception of pHNAHM7C44 (IncHI2), for which similar conjugation frequencies (Z = −0.655; *P *= 0.513 [by a Mann-Whitney U test]) were observed for both E. coli C600 and K. pneumoniae ATCC 13883 (Fig. 2).

### Comparison of the invasion and persistence of *mcr-1*-bearing plasmids in E. coli and K. pneumoniae.

**FIG 3:**
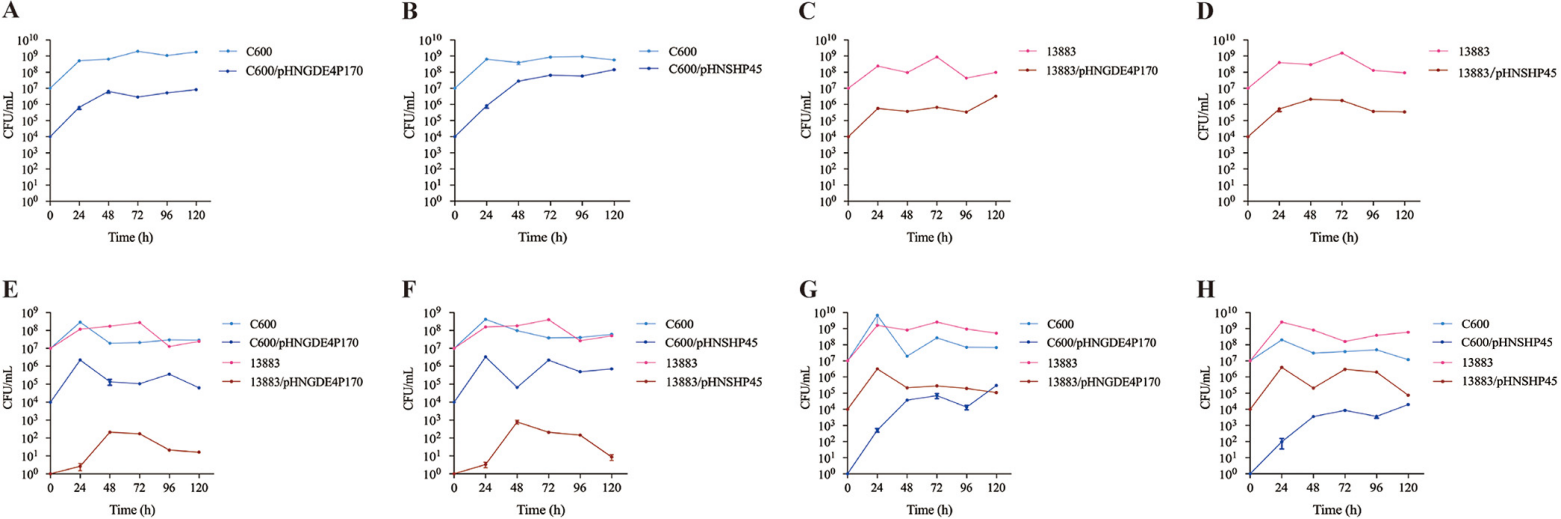
Bacterial population dynamics in cocultures with plasmid-free and *mcr-1* plasmid-containing E. coli and K. pneumoniae. (A and B) Coculture of *mcr-1* plasmid-free E. coli C600 (10^7^ CFU/mL) with 10^4^ CFU/mL of E. coli C600/pHNGDE4P170 (A) or E. coli C600/pHNSHP45 (B). (C and D) Coculture of *mcr-1* plasmid-free K. pneumoniae ATCC 13883 (10^7^ CFU/mL) with 10^4^ CFU/mL of K. pneumoniae ATCC 13883/pHNGDE4P170 (C) or K. pneumoniae ATCC 13883/pHNSHP45 (D). (E to H) Coculture of E. coli C600 (10^7^ CFU/mL) and K. pneumoniae ATCC 13883 (10^7^ CFU/mL) with 10^4^ CFU/mL of E. coli C600/pHNGDE4P170 (E), E. coli C600/pHNGDE4P170 (F), K. pneumoniae ATCC 13883/pHNGDE4P170 (G), or K. pneumoniae ATCC 13883/pHNSHP45 (H).

The invasion and persistence of mcr-1-bearing plasmids in E. coli and K. pneumoniae were examined by assessing the ability of mcr-1-carrying plasmids to invade plasmid-free populations. First, the parental strain (E. coli C600 or K. pneumoniae ATCC 13883) was cocultured with the respective *mcr-1* plasmid-carrying strain individually, and the populations with/without the plasmid were detected every 24 h following passaging. For the individual cultures, the population of the plasmid-carrying strain was stably present for a duration of 120 h (Fig. 3A to D). The competitive cocultures were constructed by coculturing an *mcr-1* plasmid-carrying strain with a population of plasmid-free bacteria of the same strain or a strain of another species (Fig. 3E to H). When *mcr-1* plasmid-carrying E. coli C600 was cocultured with plasmid-free E. coli C600 and K. pneumoniae ATCC 13883^RIF^, the population of K. pneumoniae ATCC 13883^RIF^ carrying an *mcr-1* plasmid was detected at a very low count (<10 CFU/mL) at 24 h, which further increased to the highest point of ~10^2^ to 10^3^ CFU/mL at 48 h, indicating that plasmids carrying *mcr-1* are capable of invading K. pneumoniae strains (Fig. 3E and F). Thereafter, the number of K. pneumoniae bacteria harboring *mcr-1* decreased progressively (Fig. 3E and F), indicating that K. pneumoniae ATCC 13883^RIF^ carrying an *mcr-1* plasmid has a competitive disadvantage in the coculture environment. With *mcr-1* plasmid-carrying K. pneumoniae ATCC 13883^RIF^ in the cultures containing plasmid-free K. pneumoniae ATCC 13883^RIF^ and E. coli C600, a rapid increase in the number of E. coli C600 bacteria harboring an *mcr-1* plasmid was observed over the initial 48 h, reaching 10^4.5^ CFU/mL (pHNGDE4P170) and 10^3.5^ CFU/mL (Fig. 3G and H). Importantly, the population of *mcr-1*-carrying E. coli C600 was maintained over the whole duration of 120 h (Fig. 3G and H), even outnumbering *mcr-1* plasmid-carrying K. pneumoniae ATCC 13883^RIF^ (Fig. 3G), indicating that *mcr-1*-positive E. coli exhibited a long-term competitive advantage in these competition environments. Together, these observations suggest that *mcr-1* plasmids were persisting at a higher magnitude in E. coli than in K. pneumoniae, and the latter populations harboring *mcr-1* exhibited a long-term competitive disadvantage, causing the population to gradually dwindle.

## DISCUSSION

It is widely acknowledged that plasmids are extrachromosomal genetic elements that represent common vehicles for carrying antimicrobial resistance genes (22). Although antibiotic resistance plasmids benefit the host cell in the environment where the antibiotic is present, there could also be fitness costs associated with carrying the plasmid (23, 24). In the absence of antibiotics, the fitness cost could outweigh the benefit of carrying the plasmid, leading to plasmid loss during cell division (25, 26). To investigate the factors contributing to the different prevalences of *mcr-1*-bearing plasmids in E. coli and K. pneumoniae, the plasmid stabilities and plasmid-associated fitness costs in these two species were evaluated by using the dominant *mcr-1* plasmids (IncX4 and IncI2 types). We found that *mcr-1* plasmids of the IncX4 and IncI2 types were stably maintained in both E. coli and K. pneumoniae following 35 days of passage without colistin, suggesting that plasmid stability is not responsible for the different prevalences of *mcr-1* in these two bacterial species. A further investigation was conducted on the fitness effect of carrying the IncX4- and IncI2-type *mcr-1* plasmids in these two species. The results showed that *mcr-1* plasmids of the IncX4 and IncI2 types conferred a fitness advantage on E. coli, while IncI2-type *mcr-1* plasmids imposed a slight fitness cost on K. pneumoniae (Fig. 1). Similarly, an increase in fitness with the carriage of *mcr-1* plasmids was also observed in a previous study with E. coli DH5α (27), and reduced biological fitness was observed with an *mcr-1*-carrying K. pneumoniae strain (17). MCR-1 usually imposes a fitness cost on host bacteria (15–17); however, we observed a slight fitness advantage for E. coli harboring *mcr-1* plasmids. We propose that a higher plasmid conjugation transfer efficiency in E. coli competition cultures may overcome the fitness cost of *mcr-1*-bearing plasmids (19). Overall, these data suggest that the presence of *mcr-1* can be beneficial for E. coli while being disadvantageous for K. pneumoniae, indicating that the acquisition of *mcr-1* (or *mcr-1* plasmids) comes with a fitness cost to the latter strain, reducing the competitive ability of *mcr-1*-carrying K. pneumoniae.

In addition to the above-mentioned plasmid stability and plasmid-associated fitness costs, conjugation efficiency is another key determinant affecting plasmid persistence in bacterial populations as plasmids are commonly acquired by bacteria via conjugation (13, 28, 29). With this, the frequency of conjugation of *mcr-1* plasmids of the IncX4, IncI2, IncHI2, IncP, or IncF type (natively from E. coli and K. pneumoniae donors) to recipient strains of either E. coli or K. pneumoniae was investigated. We found that the conjugative efficiency of *mcr-1* plasmids was dependent on the recipient species. E. coli as a recipient generally demonstrated a higher conjugation efficiency than K. pneumoniae, regardless of the donor species and the Inc types of the *mcr-1* plasmids (Fig. 2). This finding suggests that *mcr-1*-bearing plasmids could spread more readily among E. coli than among K. pneumoniae populations. It was previously reported that the conjugation of an extended-spectrum-β-lactamase (ESBL)-carrying plasmid was affected by a combination of three factors (plasmid and donor and recipient strains of E. coli) (30). Differences in the genetic backgrounds of E. coli and K. pneumoniae may be responsible for the different conjugation rates, but this remains to be elucidated.

Furthermore, in a coculture environment, E. coli also presented itself as an amazing recipient of the *mcr-1* plasmid from K. pneumoniae, and the *mcr-1* plasmid-carrying E. coli population was able to persist in the mixed environment (Fig. 3G and H). The population of K. pneumoniae carrying the *mcr-1* plasmid increased initially, indicating that K. pneumoniae was able to take up the *mcr-1* plasmid from E. coli during the initial coincubation. However, it decreased progressively after 48 h (Fig. 3E and F). Such a difference likely resulted from the higher frequency of conjugation of *mcr-1*-bearing plasmids in E. coli than in K. pneumoniae (Fig. 2). This opinion is supported by previous studies in which plasmids with a higher conjugation rate were able to invade bacterial populations more effectively (13, 28, 29). Also, a recent study by Yi et al. indicated that efficient conjugation was sufficient to overcome the fitness cost of *mcr-1* carriage and enhance plasmid persistence in a bacterial community (19). Overall, these observations suggest that *mcr-1* plasmids could invade E. coli more easily and be maintained in E. coli with higher stability than in K. pneumoniae (Fig. 3). Together with the fitness benefit mediated by *mcr-1*-bearing plasmids for E. coli while being disadvantageous for K. pneumoniae (Fig. 1), *mcr-1* plasmid-carrying E. coli could have outcompeted K. pneumoniae in the bacterial community, thus contributing to the high prevalence of *mcr-1* among E. coli species.

In conclusion, our study revealed that *mcr-1*-bearing plasmids were associated with a fitness advantage, higher conjugation efficiency, and greater persistence of E. coli. Altogether, these results confirm that effective conjugation and fitness benefits are crucial for the dissemination and maintenance of *mcr-1* plasmids in bacteria (19).

## MATERIALS AND METHODS

### Strains and plasmids.

Twenty-six *mcr-1*-positive *Enterobacterales* strains (E. coli, *n *= 19; K. pneumoniae, *n *= 7) obtained from various sources, including humans, swine, chickens, and fish, in China between 2014 and 2018 were selected for this study (Table 1) (6, 27). The *mcr-1* gene was carried by plasmids of different types (i.e., IncX4, IncI2, IncHI2, IncP, IncF29:A–:B–, and IncFIB) (Table 1). *mcr-1*-negative K. pneumoniae ATCC 13883, E. coli ATCC 25922, and E. coli C600 were employed as recipient strains of *mcr-1* plasmids. The pLac-eGFP plasmid, which contains an enhanced green fluorescent protein (eGFP) constitutive expression cassette, was transformed into E. coli DH5α, and the resulting strain was named E. coli DH5α-GFP. E. coli DH5α-GFP was used as a reference strain for the *in vitro* competition assay.

**TABLE 1:** Strains used in this study

Strain	Plasmid type	Plasmid name	Plasmid length (kbp)	Organism	Origin	Yr of isolation	Reference
AHM5C55	IncX4	pHNAHM5C55	~33.3	Escherichia coli	Chicken	2015	6
AHM5C65	IncX4	pHNAHM5C65	~33.3	Escherichia coli	Chicken	2015	6
SHP23	IncX4	pHNSHP23	~33.3	Escherichia coli	Swine	2016	27
GDE4P165	IncX4	pHNGDE4P147	~33.3	Escherichia coli	Swine	2014	6
GDE4P170	IncX4	pHNGDE4P170	~33.3	Escherichia coli	Swine	2014	6
AHM7C6I	IncX4	pHNAHM7C6I	~33.3	Klebsiella pneumoniae	Chicken	2017	33
AHM7C8I	IncX4	pHNAHM7C8I	~33.3	Klebsiella pneumoniae	Chicken	2017	33
AHM7C25I	IncX4	pHNAHM7C25I	~33.3	Klebsiella pneumoniae	Chicken	2017	33
AHM7C33I	IncX4	pHNAHM7C33I	~33.3	Klebsiella pneumoniae	Chicken	2017	33
AHM8C15	IncX4	pHNAHM8C65	~33.3	Klebsiella pneumoniae	Chicken	2018	This study
SHP16	IncI2	pHNSHP16	~60	Escherichia coli	Swine	2016	27
SHP45	IncI2	pHNSHP45	~64	Escherichia coli	Swine	2016	6
SHP50	IncI2	pHNSHP50	~60	Escherichia coli	Swine	2016	27
AHM7C31	IncI2	pHNAHM7C31	~60	Escherichia coli	Chicken	2017	6
AHM7C61	IncI2	pHNAHM7C61	~60	Escherichia coli	Chicken	2017	6
BJ7H48	IncI2	pHNBJ7H48	~64	Klebsiella pneumoniae	Human	2017	This study
SHP45	IncHI2	pHNSHP45-2	~250	Escherichia coli	Swine	2016	34
SHP26	IncHI2	pHNSHP26	~240	Escherichia coli	Swine	2016	27
SHP51	IncHI2	pHNSHP51	~244.4	Escherichia coli	Swine	2016	27
AHM7C44	IncHI2	pHNAHM7C44	~244.4	Escherichia coli	Chicken	2017	6
AHM8C44	IncHI2	pHNAHM8C44	~244.4	Escherichia coli	Chicken	2018	6
AHM8C65	IncHI2	pHNAHM8C65	~244.4–310.1	Escherichia coli	Chicken	2018	6
GDT6F1	IncP	pHNGDT6F1	~50.4	Escherichia coli	Fish	2016	35
GDP6F36	IncP	pHNGDP6F36	~52.7	Escherichia coli	Fish	2016	35
SHP22	IncF29:A−:B−	pHNSHP22	~78.0	Escherichia coli	Swine	2016	27
HZ6P5	IncFIB	pHNHZ6P5	~190.0	Klebsiella pneumoniae	Swine	2016	36

### Development of rifampicin resistance by serial passages.

The rifampicin-resistant K. pneumoniae ATCC 13883^RIF^ strain was obtained by serial passaging of rifampicin-sensitive K. pneumoniae ATCC 13883 over a period of 8 days. Briefly, a culture of K. pneumoniae ATCC 13883 grown overnight was diluted to 10^6^ CFU/mL, of which 100 μL was plated onto Luria-Bertani (LB) agar containing rifampicin (0.5× MIC). Following a 24-h incubation at 37°C, viable cells were resuspended in fresh LB broth, and a 100-μL bacterial suspension containing 10^6^ CFU/mL was plated onto LB agar containing rifampicin (1× MIC). The concentration of rifampicin increased exponentially in serial passages. After obtaining rifampicin-resistant K. pneumoniae, the stability of rifampicin resistance in K. pneumoniae was further assessed by daily serial passages of the culture without antibiotics for 6 days. The resistance of K. pneumoniae ATCC 13883^RIF^ to rifampicin was stable (MIC of rifampicin of >512 mg/L) (see Table S2 in the supplemental material), and we then stored K. pneumoniae ATCC 13883^RIF^ in 25% glycerol stocks containing 500 mg/L rifampicin.

### Antimicrobial susceptibility test.

The MICs were determined using the broth microdilution method for streptomycin, rifampicin, and colistin according to Clinical and Laboratory Standards Institute (CLSI) guidelines (10). E. coli ATCC 25922 was used as the reference strain.

### Transformation experiments and plasmid stability.

The *mcr-1* plasmids were isolated from native K. pneumoniae strains (pHNAHM7C25I [IncX4] and pHNBJ7H48 [IncI2]) and E. coli strains (pHNGDE4P170 [IncX4] and pHNSHP45 [IncI2]) using the Qiagen plasmid midi kit. These plasmids were introduced into the recipient strains K. pneumoniae ATCC 13883 and E. coli C600 by electroporation. The bacterial cells were plated onto LB agar containing 4 mg/L colistin to select for those with successful transformation of the *mcr-1* plasmid, and further confirmation was conducted by PCR with primers *mcr-1*-F (5′-TCCAAAATGCCCTACAGACC-3′) and *mcr-1*-R (5′-GCCACCACAGGCAGTAAAAT-3′).

The stability of the *mcr-1* plasmids in E. coli C600 and K. pneumoniae ATCC 13883 was evaluated as reported previously (31). Briefly, a single colony was selected and grown overnight in 3 mL LB broth at 37°C with shaking at 180 rpm. Serial passages were conducted daily by inoculating 3 μL of a culture grown overnight into 3 mL LB broth for a total duration of 35 days. Every 3 or 7 days, bacterial cultures were collected and plated onto antibiotic-free MacConkey agar. One hundred colonies were randomly selected for detecting the presence of *mcr-1* plasmids by PCR. Plasmid retention was calculated as the percentage of *mcr-1*-carrying colonies among the 100 selected colonies. The assay was conducted in biological triplicates.

### Competition experiments *in vitro*.

The fitness of E. coli C600, K. pneumoniae ATCC 13883, and their corresponding transformants carrying the *mcr-1* plasmid was measured in a competition assay against a reference strain, E. coli DH5α-GFP (17). Briefly, a single colony was selected and grown overnight in M9 minimal medium supplemented with the appropriate antibiotics. The culture grown overnight was diluted 1:100 in fresh LB broth. The experimental and reference strains were mixed at a ratio of 1:1, followed by a 24-h incubation at 37°C. At 0 and 24 h, the bacterial culture was collected, diluted in phosphate-buffered saline (PBS) at a 1:100 dilution, and analyzed using flow cytometry. The formula RF = [ln(E/R)*_t_* − ln(E/R)_0_]/*T* was used to verify the relative fitness (RF), where E/R is the ratio of the tested experimental strain over the reference strain E. coli DH5α-GFP and *T* represents the generation. The assay was conducted in biological triplicates.

### Bacterial conjugation assays.

The plasmid conjugation efficiencies of *mcr-1*-carrying E. coli and K. pneumoniae strains were investigated as described previously, with minor modifications (32). Briefly, 26 native E. coli and K. pneumoniae strains carrying *mcr-1* plasmids were used as the donor strains (Table 1), while E. coli C600 or K. pneumoniae ATCC 13883^RIF^ was employed as the recipient strain. Transconjugants of E. coli C600 were selected on LB agar supplemented with 2,000 mg/L streptomycin and 4 mg/L colistin, whereas transconjugants of K. pneumoniae ATCC 13883^RIF^ were selected on LB agar containing 200 mg/L rifampicin and 4 mg/L colistin. The presence of the *mcr-1* plasmid was further confirmed by PCR. The conjugation frequency was calculated as the ratio of transconjugants over recipient cells.

### Plasmid invasion assays.

The plasmid invasion assay was performed as described previously, with some modifications (18). A single colony was inoculated into LB broth and incubated at 37°C overnight with shaking at 180 rpm. The culture of the plasmid-free strain (E. coli C600 or K. pneumoniae ATCC 13883^RIF^) grown overnight was diluted at a 1:100 ratio into 2 mL fresh LB broth and mixed with 1:100,000 dilutions of *mcr-1* plasmid-carrying strains (E. coli C600/pHNGDE4P170, E. coli C600/pHNSHP45, K. pneumoniae ATCC 13883^RIF^/pHNGDE4P170, or K. pneumoniae ATCC 13883^RIF^/pHNSHP45) grown overnight. The mixed culture was incubated at 37°C with a low shaking speed (80 rpm). The mixed culture was passaged in fresh LB broth at a 1:100 dilution every 24 h for a duration of 120 h. Cultures were collected at the end of each 24-h passage (24, 48, 72, 96, and 120 h) for the counting of viable cells using the appropriate antibiotic-containing LB agar (Table S3).

### Statistical analyses.

Statistical analysis was performed using Prism 9 and the IBM SPSS Statistics 21 program. One-way analysis of variance with Benjamini-Hochberg correction was used to determine the statistical differences in the relative fitnesses of bacteria among the five groups. The nonparametric Mann-Whitney U test was used for comparisons of differences in the conjugation frequencies of plasmids between two groups. The statistical significance threshold was set to an α value of 0.05.
