# Supplementary material for: Greater Invasion and Persistence of mcr-1-Bearing Plasmids in Escherichia coli than in Klebsiella pneumoniae
Source: Microbiol Spectr. 2023 Mar 28;11(2):e03223-22. doi: 10.1128/spectrum.03223-22 (PMC10100767; doi:10.1128/spectrum.03223-22)
Supplement: Supplemental file 1 — Tables S1 to S3. Download spectrum.03223-22-s0001.pdf, PDF file, 0.2 MB [file spectrum.03223-22-s0001.pdf]

Table S1. The stability of *mcr-1*-carrying plasmids in *E. coli* and *K. pneumoniae*

Strains	Species	Plasmid type	%stability							
			1d	3d	5d	7d	14d	21d	28d	35d
C600/pHNGDE4P170	*E. coli*	IncX4	100	100	100	100	100	100	100	100
C600/pHNAHM7C25I	*E. coli*	IncX4	100	100	100	100	100	100	100	100
C600/pHNSHP45	*E. coli*	IncI2	100	100	100	100	100	100	100	100
C600/pHNBJ7H48	*E. coli*	IncI2	100	100	100	100	100	100	100	100
13883/pHNGDE4P170	*K. pneumoniae*	IncX4	100	100	100	100	100	100	100	100
13883/pHNAHM7C25I	*K. pneumoniae*	IncX4	100	100	100	100	100	100	100	100
13883/pHNSHP45	*K. pneumoniae*	IncI2	100	100	100	100	100	100	100	100
13883/pHNBJ7H48	*K. pneumoniae*	IncI2	100	100	100	100	100	100	100	100

Table S2. The resistance of *K. pneumoniae* ATCC 13883^{RIF} to rifampicin.

Strains	MIC of rifampicin (mg/L)						
	12 h	24 h	48 h	72 h	96 h	120 h	144 h
K. pneumoniae ATCC 13883^{RIF}	> 512	> 512	> 512	> 512	> 512	> 512	> 512

Table S3. Viable counting of the strains using LB agar with different antibiotic selection.

Strain	Viable count obtained from
E. coli C600	LB agar with streptomycin – LB agar with streptomycin+colistin
E. coli C600 with *mcr-1* plasmid (pHNGDE4P170 or pHNSHP45)	LB agar with streptomycin+colistin
K. pneumoniae 13883^{RIF}	LB agar with rifampicin – LB agar with rifampicin+colistin
K. pneumoniae 13883^{RIF} with *mcr-1* plasmid (pHNGDE4P170 or pHNSHP45)	LB agar with rifampicin+colistin
